# Overdose beliefs and management practices among ethnic Vietnamese heroin users in Sydney, Australia

**DOI:** 10.1186/1477-7517-6-6

**Published:** 2009-04-27

**Authors:** Lisa Maher, Hien T Ho

**Affiliations:** 1 National Centre in HIV Epidemiology and Clinical Research, Sydney, Australia; 2 School of Public Health and Community Medicine, University of New South Wales, Sydney, Australia; 3 Hanoi School of Public Health, Hanoi, Vietnam

## Abstract

**Background:**

Ethnic Vietnamese injecting drug users (IDUs) in Australia draw on a range of beliefs and etiologic models, sometimes simultaneously, in order to make sense of health and illness. These include understandings of illness as the result of internal imbalances and Western concepts of disease causation including germ/pollution theory.

**Methods:**

Observational fieldwork and in-depth interviews were conducted between 2001 and 2006 in neighbourhoods characterised by high proportions of Asian background IDUs and street-based drug markets. Eligibility criteria for the study were: 1) ethnic Vietnamese cultural background; 2) aged 16 years and over and; 3) injected drugs in the last 6 months.

**Results:**

Participants commonly attempted to treat heroin overdose by withdrawing blood (rút máu) from the body. Central to this practice are cultural beliefs about the role and function of blood in the body and its relationship to illness and health. Participants' beliefs in blood were strongly influenced by understandings of blood expressed in traditional Chinese and Vietnamese medicine. Many participants perceived Western drugs, particularly heroin, as "hot" and "strong". In overdose situations, it was commonly believed that an excessive amount of drugs (particularly heroin) entered the bloodstream and traveled to the heart, making the heart work too hard. Withdrawing blood was understood to reduce the amount of drugs in the body which in turn reduced the effects of drugs on the blood and the heart.

**Conclusion:**

The explanatory model of overdose employed by ethnic Vietnamese IDUs privileges traditional beliefs about the circulatory, rather than the respiratory, system. This paper explores participants' beliefs about blood, the effects of drugs on blood and the causes of heroin overdose in order to document the explanatory model of overdose used by ethnic Vietnamese IDUs. Implications for overdose prevention, treatment and management are identified and discussed.

## Introduction

Opioid overdose is the leading cause of premature death among heroin users [1]. A meta-analysis of longitudinal studies of opioid users found a mortality rate 13 times greater than age and gender-matched peers [2]. Major risk factors for opiate fatalities include being an older, single unemployed male, having a history of heroin dependence, not being in drug treatment and concomitant consumption of alcohol and/or benzodiazepines [3,4]. Systemic disease, in particular, hepatic and pulmonary disease, may account for the strong age and gender patterning of overdose fatalities [5]. While the majority of overdose deaths occur with witnesses present and death is not immediate in most cases [6,7], the opportunities this presents for effective intervention are not always realized, with one study reporting no intervention prior to death in 79 percent of fatal cases [8].

Several factors appear to place ethnic Vietnamese injecting drug users (IDUs) at increased risk of fatal opioid overdose [9]. Most participants in the current study were single, unemployed males aged in their late 20s and early 30s, with a history of recent imprisonment. These are factors previously identified as being associated with fatal overdose [1,4]. Secondly, high rates of concomitant alcohol and benzodiazepine use, which increase the risk of opioid overdose, have also been identified in this group [10,11]. Thirdly, unlike opioid overdose cases nationally which occur in private settings, opioid overdoses in the study site (South Western Sydney or SWS) typically occur in public settings [12]. While in theory, the prospect of successful intervention is higher when overdoses occur in public, ethnic Vietnamese IDUs are often reluctant to seek help due to fear of police attendance [13].

Vietnamese IDUs in Australia are a marginalised, socially isolated and economically disadvantaged group characterised by high levels of drug-related harm [14]. Studies have documented high rates of blood-borne viral infection and associated risk behaviours [15-17], contact with the criminal justice system and incarceration [18] and heroin related mortality [9], while noting limited contact with health services [10,15] and poor treatment outcomes [19,20]. Culture provides a lens through which people interpret, understand and respond to the worlds they live in. Following Kleinman [21], we sought to explore beliefs about blood, the effects of drugs on blood and the causes of heroin overdose in order to document the explanatory model of overdose used by ethnic Vietnamese IDUs in Sydney, Australia. By providing insights into how cultural factors are interwoven with everyday experiences of injecting drug use and explanatory models of overdose, our approach illustrates significant differences between emic and biomedical explanatory models. Understanding the nature of these differences has important implications for prevention interventions designed to reduce overdose-related mortality and morbidity in this population.

## Research methods

As part of an ongoing program of research focusing on injecting drug use and related harms in culturally and linguistically diverse communities [22,23] the current study was designed to explore the impact of cultural beliefs and practices on risk taking and health seeking behaviours among ethnic Vietnamese IDUs [24]. Observational fieldwork and in-depth interviews were conducted between 2001 and 2006 in neighbourhoods characterised by high proportions of Asian background IDUs and street-based drug markets. Eligibility criteria for the study were: 1) ethnic Vietnamese cultural background; 2) aged 16 years and over and; 3) injected drugs in the last 6 months. Ethical approval for the project was granted by the University of NSW Humans Research Ethics Committee and participants were reimbursed $AUD20.

Participants were recruited using a mix of theoretical [25] and snowball sampling strategies [26] drawing on street and social networks and previous research contacts. Interviews were conducted on the street, in restaurants, bars and coffee shops and in private homes. Interviews were tape-recorded and transcribed and, where indicated, translated into English. A total of 64 in-depth interviews were conducted: 28 in English, 30 in Vietnamese and six in a mixture of English and Vietnamese. Data analysis was conducted simultaneously with data collection and data interpretation and was iterative throughout the research process. Open coding was used to inductively classify data into initial categories or themes, followed by axial coding to examine the data for regularities and variations within and between themes [27].

## Results

### Beliefs about blood

Vietnamese consider blood (*huyết *in Vietnamese) as essential to life [28]. Dong Y^1 ^holds that Qi (*khí *in Vietnamese), blood and body fluids are the most fundamental elements of the human body and life arises from the metabolism of these substances [28]. Participants expressed the main function of blood as "keeping life".

Blood is in your body and gives life to all the parts in your body (Binh, 24 year-old male).

Blood is very important, because it carries oxygen to and from the heart... Keep your life (Yen, 26 year-old female).

Qi and blood are closely related in a Yin-Yang relationship and are often collectively referred to as two attributes of the one thing [*khí huyết*]. Health is viewed as the product of the relationship or harmony between Qi and blood. Qi creates and controls the movement of blood, while blood nourishes Qi [28,29]. Blood was viewed by participants as keeping the organs within the body functioning well by balancing and harmonising [*điều hoà*] the body and helping to fight sickness and disease.

Blood circulates in the body. Qi and blood is balanced and harmonised (Thanh, 37 year-old male).

Blood is to balance and harmonise your body. For instance, if blood balances and harmonises well, you don't have sickness or pain (Dao, 36 year-old male).

Participants spoke at length about the characteristics of blood and, in particular, the features that defined "good" blood and "bad" blood. Blood was typically characterised in terms of colour and viscosity. Participants considered blood to be good [*máu tốt*] when it was clean, bright red, and not too thick or thin. When blood is thin, it is able to circulate easily, carrying essential nutrients and oxygen throughout the body. Conversely, blood is believed to be "bad" (*máu xấu*) when it is dark and thick. According to Dong Y, heat increases the viscosity of blood as it absorbs body fluids and slows down the flow of blood in the vessels.

Thin blood is the best. It should be real red – is the good one. Dark blood no good, like burgundy. Thickness is no good. I saw my friend is in the army and from his experience he told me and he have thin blood and very healthy. He never takes alcohol or any drugs (Thang, 33 year-old male).

If blood is too thick, it cannot run or flow properly, which may have negative effects on health. This is consistent with traditional Vietnamese and Chinese medicine where the flow of blood represents the circulation of Qi in the body [28].

Thin is better than thick. Thick blood is very bad because it is so dangerous for health that it can make you feel dizzy. Thick blood goes slowly, but the thin goes quick (Thanh, 47 year old male).

Both colour and viscosity are affected by different factors including diet and fluid intake. Some participants believed that drinking lots of fluid, particularly water, would clean and thin the blood, facilitating its circulation in the body. These beliefs about the significance, function and characteristics of blood, which have their roots in Dong Y, informed a specific set of beliefs about the impact of drugs on the blood.

All participants felt that drug use, especially heroin use, had a negative effect on the body and, in particular, on blood. Drugs were widely believed to be responsible for "bad blood" with most participants believing that using drugs made their blood thick [*máu đặc*], dark [*máu đậm*] and dirty [*máu dơ*]. Participants also felt that as drugs were "hot" [*máu nóng*] they heated the blood and raised the body's temperature and heart rate.

Participants felt that once they were addicted to heroin, their blood needed the drug in order to function. This is a very specific concept of dependence or addiction. Within the biomedical literature, dependence is typically defined as a syndrome comprising both physiological and psychological dependence [1]. The international classification system of the DSM-IV combines the criteria for both abuse and dependence into a single set of diagnostic criteria and requires the presence of either tolerance or withdrawal [30]. In contrast, for ethnic Vietnamese IDUs, dependence may also manifest in the blood. Participants frequently referred to their blood as being "addicted" or needing drugs.

You can't do anything about it without them cause your blood need it [heroin]. When you use it, your blood needs it and requires it all the time (Hai, 42 year-old male).

Dependent heroin use is believed to alter the natural balance of the body with the blood system requiring heroin in order to function. Drugs, particularly heroin, were widely believed to be responsible for "bad blood" and for making blood thick and dark.

[W]hen you use drug or a lot of it, your blood is darker than other healthy person whose blood is bright red, normal red (Yen, 26 year-old female).

Blood of players [drug users] is darker and thicker. If you are not playing, your blood would be fresh red and thinner (Minh Khoi, 35 year-old male).

Detoxification or the process of withdrawing from heroin or "hanging out" (a state described by participants as "*bị vã*") was identified as making the blood even thicker.

Because when you are hanging out for two or three days, your blood will be thickened. Thin is better than thick. Thick is very bad as it is very dangerous for health (Thanh, 37 year old male).

When we hang out, the blood is really dark and thick as candy (đặc kẹo) (Hue, 23 year-old female).

Beliefs about the effects of drugs on the blood are also consistent with beliefs about the need for balance [*điều hoà*] within the body. Most participants reported feeling unwell or unbalanced both when using drugs heavily and when withdrawing from them. In both situations, participants described their blood as being dark and thick. They believed that when balance was restored (whether through not using drugs or not withdrawing from them), blood returned to normal.

Heroin was also believed to make the blood warmer and flow more smoothly within the circulatory system. On the other hand, when people are withdrawing or hanging out, blood is believed to become cold and stagnate within the vessels.

This thing [heroin] is too hot, like Western medicine (Tuan, 44 year old male).

The white stuff is really hot. It makes the blood thinner, it increases your body temperature and it makes the blood circulate. If you don't have that stuff, our body temperature will drop, and the body is not functioning. Because our body used to drugs. We need that stuff to thin our blood, to make it flow and circulate. That's how your body fight against the cold. If we don't have it, our blood will hang out. It will go dark and thick. It slows the blood down, blood flows slowly so our body feels weak. We got no energy to fight back. Other people might feel cool but you have to wear two, three shirts and still feel cold. It's not cold from the outside but cold from the inside (Nhat, 41 year-old male).

Traditional Vietnamese beliefs hold that Western medicine, and antibiotics in particular, are often too hot and sometimes too strong for Vietnamese people [31]. Some participants attributed heroin with the "hot" properties of Western medicine.

Heroin makes your blood hot. It's like taking Western medicine, it makes your blood hot because it has a lot of chemicals in it. Because most of the Western medicine, after you take it, your body feels hot (Nam, 24 year-old male).

In Dong Y, the temperature of blood is believed to influence its circulation. If blood is too hot, bleeding may result because the heart speeds up the flow of blood. In contrast, cooling of the blood may impede flow causing blood stasis [29]. Participants also referred to the effects of temperature on circulation of blood within the context of drug use. They felt that using drugs made the body and the blood hot which increases the flow of blood within the cardiovascular system. According to participants, this excess heat also produces "hot" symptoms such as skin eruptions and constipation. These accounts are also consistent with traditional beliefs which view skin eruptions and constipation as symptoms of a hot or unbalanced body [28].

[What does heroin do to the blood?] Make the blood hot man. Yeah, make your face turn red, you know, make you sweat a lot. You know, sometimes you get pimple you know, yeah. [Do you have pimples before?] No, no. But when I'm using, yeah. I can't shit (Phi, 39 year-old male).

Many participants also believed that drugs made the blood "dirty". Street drugs such as heroin were described as "dirty" and "unpure". Participants widely believed that heroin was mixed with a ranged of substances including fillers (e.g. sugars), other pharmaceuticals and toxic chemicals. When heroin is injected, these impurities are believed to enter the bloodstream and contaminate the blood.

Heroin of course is not clean. It's like this. When they got the drugs from the opium plants, the dealers mix it with this stuff, that stuff. Not only their hands are dirty but like you have one kilogram [of heroin] you want to make one and a half kilograms you have to put all different stuff in. That's how they make money (Au, 33 year-old male).

This section has explored participants' beliefs about blood, the characteristics that define good and bad blood, and the effects of drugs on the blood. Importantly, participants' understandings were shaped by traditional beliefs about blood and Western medicines, which inform the explanatory model of overdose discussed below.

### Explanatory model of overdose

#### Causes of overdose

Most participants believed that overdose occurred as a result of people using too much or "over their dose". Participants recognised that even relatively small amounts of heroin could result in overdose, the key being that these amounts exceeded their "normal" dose.

[We overdose] because we play over our dose (Hai, 42 year-old male).

Overdose is because you use too much drugs over your normal limit. You can only use one dose but you use three doses and you overdose (Minh, 42 year-old male).

While participants acknowledged that tolerance varied from person to person, almost all spoke of having a "normal dose" or limit. This is similar to the concept of tolerance described in the biomedical literature which is characterised by a need for markedly increased amounts of the substance in order to achieve intoxication or the desired effect and/or markedly diminished effects with continued use of the same amount [1]. Participants also implicated periods of abstinence or infrequent use in reducing tolerance and placing users at increased risk of overdose [32].

He got locked up for a year. And he came out, using that same amount as he used when he got locked up. And he didn't know that his dose, his tolerance went right down cause he's been in jail for a whole year. He OD'd. He died you know (Thuan, 22 year-old male).

The reason of overdose is they use over their dose then they stop for a while, and [then] they play again. Before their dose is really high. They think it's still the same, but their dose is not the same. We quit for a while our body goes back to normal (Nhat, 41 year-old male).

Even short periods of abstinence such as attempts at detoxification or withdrawal can reduce tolerance. Participants noted that when people were "hanging out" they were weak and unable sometimes to tolerate using the amount they normally used. This group was viewed as particularly vulnerable to overdose.

Normally people think they have a shot, a quarter, and they think [it's] just a quarter. But when they hang too much and their body is weak so they put in a quarter and, you know, your body can't take it (Huyen, 21 year-old female).

According to participants, many heroin users accidentally overdose because they want to get "more stoned". Some participants reported deliberately using "over their dose" in order to intensify the pleasurable effects of heroin.

Overdose is because they use over their dose. In the case that the person is greedy, they think to play to get stoned but they didn't think that their body couldn't handle it so they overdose (Huy, 42 year-old male).

Participants repeatedly identified "being greedy" as a major cause of overdose in heroin users. Beliefs about the role of "greed" in the etiology of overdose are consistent with biomedical interpretations which hold that overdose occurs when drug concentration in the body exceeds the user's tolerance. However, because heroin is an illegal drug, its manufacture, sale and consumption are largely unregulated. There are no product warnings and the potency of each dose is unknown. Many participants felt that fluctuations in heroin purity contributed to overdoses.

The quality of all the dealers is different ... They mix it to make it bigger when they sell it. They didn't know, they mix it with this stuff, that stuff, so they can compress it. I know, I used to do it. For example, I buy five ounces of pure heroin, I will mix forty percent of heroin and sixty percent of sugar and sleeping pills. If I get five ounces of pure white with cheap price, I will put sixty percent heroin and forty percent mixture so that my gear is good. But when it's dry, I mix a lot with other substances but using less heroin so the gear is shit ... when you go to a different dealer and the gear is stronger you don't know. You still use all at once instead of two times for the thirty dollars worth. Then it would be too heavy for your dose and you OD (Lam, 24 year-old male).

However, research suggests that the adulteration of street drugs happens is infrequent and that this rarely, if ever, occurs with dangerous substances [33,34]. In Australia, a chemical analysis of 88 street-level heroin samples failed to find evidence of harmful additives or diluents [23]. The absence of toxic contaminants in this sample from a major drug market during a period of high overdose mortality suggests that harmful adulterants play an insignificant role in opioid overdose deaths in Australia [23].

Concomitant benzodiazepine and alcohol use have also been identified as risk factors for opioid overdose [1,5,35]. An examination of coronial files in NSW found that 45 and 27 percent tested positive for alcohol and benzodiazepines respectively, suggesting that many opioid-related fatalities are polydrug overdoses [36]. This was acknowledged by some participants.

Because that two strong types [heroin and sleeping pills] combine, your body can't take it. For example, you take five sleeping pills then you have a shot, you might die (Khiem Nhan, 31 year-old male).

Many participants reported using sleeping pills, either to assist when trying to quit heroin use or to manage withdrawal symptoms. Some also reported using benzodiazepines to increase the effects of heroin, potentially allowing them to reduce the quantity and cost of the heroin they used.

In summary, participants perceived using "over the dose" as the main cause of heroin overdose. They also identified "greed", the illegal, unregulated nature of the drug market and concurrent benzodiazepine use as contributing to overdose. Understandings of the actual mechanisms of heroin overdose are presented below.

#### Mechanisms of overdose

The biomedical explanatory model of fatal opioid overdose identifies opioid-induced depression of respiratory function resulting in hypoxia and death as the primary mechanism of overdose [1,4]. While respiration is affected by the lungs and associated musculature, function is dependent on external input from the CNS. Control of breathing is primarily located in the brain stem where the overall effect of opioid use is to depress neuronal activity, resulting in decreased sensitivity to changes in concentrations of oxygen and carbon dioxide outside normal ranges [35].

Ethnic Vietnamese IDUs held a very different set of beliefs regarding the underlying causal mechanisms of heroin overdose. These beliefs are grounded in specific beliefs about blood and the effects of drugs on the blood and the body. Participants identified the circulatory or cardiovascular system and not the CNS as the main bodily system implicated in heroin overdose. Within this interpretive framework, overdose occurs when the amount of drugs in the blood is too high or "over the dose". High doses of heroin place the heart under increased strain as it works to circulate the blood in order to overcome or "balance" the effects of the drug. Some participants also described how in overdose situations, drugs "hit the blood" making it so "bad" or "thick" that it can no longer circulate.

According to participants, parenteral administration of heroin results in the drug entering the blood stream directly and from there flowing straight to the heart. Thus the heart was seen as the organ first and most affected by intravenous heroin use. This is consistent with results from a related survey [11] where 60 percent of participants identified the heart as the part of the body most affected by overdose. Intravenous administration of drugs was felt to place the heart and the veins under considerable stress.

If you use over the tolerance, the blood system, it start circulating too fast. You know what I mean? And every time it go too fast it have to get through your heart and your heart start pumping too hard. Boom boom boom boom. Just your heart [is affected]. It go fast, then fast, fast. Then it go slow, slow. Then you die (Thang, 33 year-old male).

Participants offered similar understandings of the causal mechanisms underlying overdose: drugs injected into the vein travel directly to the heart, causing the heart rate to increase resulting in increased circulation of the drug-exposed blood throughout the body.

The heart is affected by overdose cause it's in the blood, that's why, it is in the blood. It goes to your heart then it goes to the whole body. For sure, it makes the heart pump too fast and you can't handle. That's why you die (Tung, 22 year-old male).

Most participants believed that in the case of overdose, the heart beats so fast that the blood cannot circulate normally. After a while, the heart is believed to stop beating because it can no longer handle the effects of excess drugs on the body.

Maybe because you shoot up too much. The heroin stimulates you. Your heart start beating fast, you blood stream gets blocked then your heart stop beating (Khiem Nhan, 31 year-old male).

In contrast to the biomedical literature where the symptoms of opioid overdose include impaired respiratory function, weak pulse and low blood pressure [35], participants identified an increase in heart rate as the primary symptom of imminent overdose. Many participants believed that during overdose, the heart has to work harder to "pump the blood". This was described as making the blood (containing the excess drugs) "over-circulate".

Overdose is over-circulating drug in the blood. Make your heart pump blood too fast and make your vein explode and you gone (Thang, 33 year-old male).

### *Rút máu*: An emic response to overdose

[What cause overdose?] Heroin, because you put too much drug in your body. So your heart can't handle it. That's why you may overdose. Too much drug, very strong. Make the heartbeat go faster, and when it goes faster, they might break the vein. You know. So that's why you die from overdose. So that's why you have to withdraw. Because we have to pull out the blood to make, you know, all the drug comes out too (Trung, 29 year-old male).

The practice of withdrawing blood [*rút máu*] in the event of heroin overdose (see additional file 1) is widespread among Vietnamese IDUs. Almost all participants in the ethnographic study were aware of this practice and 38 percent of survey participants indicated that they would withdraw blood in the event of an overdose. The practice involves withdrawing blood with a syringe, usually as soon as possible following onset.

When my friend overdosed, I withdrew blood first. Withdrew immediately, until they wake up. Usually two, three syringes of blood. It's like something is too full, you withdraw to take some of it out. I tried to pull out blood, they woke up. No need to call ambulance, just wait for a while they will wake up (Lam, 24 year-old male).

Beliefs in the efficacy of blood withdrawal were based on its status as an established folk practice and most participants had direct experience of either performing the procedure or watching it being performed by others.

He got needle out and just knock off, fall back and not wake up. I don't know how to use the needle or how it work. We was really panicking because we don't want he die in the flat. Someone is suggesting that to suck whatever back and he may be awake. I remember when I am kid and sick and my grandmother cut my head to take the blood out and it work. I grab the razor we use to cut the gear and cut his vein and squeeze the blood out. We just full on operation him. He not wake up so we take him out and put him in the stairway and call the ambulance. At that time didn't call him as OD-er. I call him as over-user. That's why at that time I think I take some back out, stop him over-sleeping (Truc, 27 year-old female).

For most participants, witnessing the recovery of overdose victims following the procedure confirmed that withdrawing blood was an effective treatment. However, several participants also had experiences where despite withdrawing blood, the person had died. These deaths were not attributed to the ineffectiveness of the antidote but rather to the person using too much.

I believe that because she's [friend] done that [died], she had too much. I believe that she had too much. That for someone to withdraw what they've taken out is, just, [to] take out like a bit (Hue, 23 year-old female).

In such cases participants believed that the person had died because they had taken an extremely high "dose" of heroin and it was impossible to withdraw the amount of blood necessary to reduce the effects of the drug.

And you know what? He still get you know, the white stuff, bubble coming out, you know like that ...biting tongue, like that. So me, my friend get the fit, two ml and one ml, you know, we take it out OK?... Twenty-one fit, take it out, all his blood! Me and another four friends... fucking he still die and I don't know why (Phi, 39 year-old male).

The practice of withdrawing blood is informed by the belief that drugs primarily affect the cardiovascular system, especially the blood and the heart. Participants strongly believed that because drugs are "in the blood" and their effects concentrated in the cardiovascular system, withdrawing blood reduces the amount of drugs in the bloodstream.

After you have a shot, the white's still in your blood system so you suck the blood out, take the drug out and reduce amount of drug in the blood (Nam, 24 year-old male).

Because participants believed that overdose increased the strain on the heart, making it beat too fast, they believed that when blood was withdrawn, there was less pressure on the heart, allowing it to beat more slowly and to circulate the remaining blood more effectively.

Suck and reduce the blood to make the tube [blood vessels] go clear. Because the blood goes too fast. It rushes down so we have to suck it out. It's like a balloon, you blow up too big, it will blow up easily (Nhat, 41 year-old male).

Most participants clearly believed that drugs made the heart work harder and faster. In the event of overdose, withdrawing blood is believed to help by reducing the effects of the drugs on the circulatory system.

If we use over the dose, the amount is too high, it makes our blood go really fast. If our heart squeezes sixty beats in one minute, it might go over sixty beats when you put the stuff in. It's like a container on top that leads to a lot of tubes underneath. But if the container pours down too fast, it will get stuck and blocked and it got nowhere to go, it will rip the vein and you will die. If we make it flow back to normal, your body will wake up (Nhat, 41 year-old male).

### Barriers to seeking medical assistance

It should be noted that, in the event of overdose, the help-seeking process is hierarchical and reflects help seeking for other health problems experienced by ethnic Vietnamese IDUs where self-managed care is the preferred option and formal medical assistance often a last resort [24]. The biggest risk incurred by withdrawing blood is in delaying biomedical treatment. The focus on immediate intervention may prevent or delay seeking medical assistance.

We withdraw blood first. If not awake, we then call the ambulance (Thanh, 37 year-old male).

I used to do [withdraw blood] to my friend, you know, a lot here. Yeah. They died. One of my friend like that, Tran, died. [After injecting heroin] Tran dropped. Yeah, he drop straight away .... We take out his blood. And you know what, we left him there. What we do? We go nightclub. We come back and he dead (Phi, 39 year-old male).

Most participants reported that they were reluctant to call an ambulance when someone overdosed due to fear of police attendance and the potential for drug-related arrests and criminal charges. Withdrawing blood was believed to provide a quick, effective antidote to overdose that enabled the victim and others present to avoid this risk. In addition to fear of police attendance, participants also identified fear of being blamed by the victim for withdrawal symptoms following the administration of Naloxone. Some participants had direct experience of precipitated withdrawal following the administration of Naloxone which often results in a strong need for heroin in order to alleviate symptoms. This may place overdose victims at risk of subsequent overdose.

When we wake up, you feel sad. They shoot up to make them feel good ... Because they are hanging out. They feel cold and uncomfortable (Nhat, 41 year-old male).

The fear of precipitated withdrawal following Naloxone administration also serves to rationalise beliefs in withdrawing blood as an antidote to overdose. If participants respond to the intervention, the need to call an ambulance is avoided and potential withdrawal averted.

If that person has it [heroin] and that person OD and you do it straight away, you might suck some of it out. But if you leave it for too long, it's in your body too much, you might not get that same blood. Blood circulates. If you leave it too long, you might not get that part of blood where the drugs are. It might be too late (Thuan, 22 year-old male).

All but three participants believed in the efficacy of withdrawing blood in the event of overdose. Many participants reported that they would only call an ambulance if withdrawing blood failed to result in a visible improvement.

After withdrawing two syringe of blood and it doesn't improve, we have to call ambulance to cure them. Because the ambulance they have medication to inject to people that makes the drugs in your body dissolve. If we don't call ambulance in that case, they will die for sure (Thanh, 37 year-old male).

Beliefs in the efficacy of this particular form of early intervention may mean that participants delay or postpone seeking medical assistance in favour of withdrawing blood as an immediate response to overdose. Sometimes, this call is too late. Participants' strong beliefs in the efficacy of withdrawing blood to treat overdose at best delays, and, at worst, prevents, ethnic Vietnamese IDUs from seeking medical assistance.

## Conclusion

This study examined ethnic Vietnamese IDUs' beliefs about blood, the effects of drugs on blood and the causes of heroin overdose. Taken together, these beliefs constitute an emic explanatory model which provides the logic for the practice of withdrawing blood (rút máu) in the event of overdose. Participants' beliefs in blood were strongly influenced by the concept of blood expressed in Dong Y. Many participants perceived Western drugs, particularly heroin, as "hot" and "strong". In overdose situations, it was commonly believed that an excessive or intolerable amount of drugs entered the bloodstream and travelled to the heart, making the heart work too hard. Withdrawing blood was understood to reduce the amount of drugs in the body which in turn reduced the effects of drugs on the blood and the heart. Hence, the explanatory model of overdose employed by Vietnamese IDUs in the current study privileges traditional beliefs about the circulatory rather than the respiratory system which underpins the biomedical explanatory model of overdose.

The majority of participants in the current study came to Australia as "boat people" from the south of Vietnam. The practice of withdrawing blood in response to overdose has not been observed in Hanoi and it may be that the explanatory model identified here, with its emphasis on the role of blood and Qi, is restricted to southern Vietnamese immigrants. However, it is important to note that the qualitative data presented here are consistent with the results of a related survey [11]. Survey participants (n = 108) believed that drugs made the blood dirty and dark in colour (53%). Participants identified the main causes of overdose as using too much or being "greedy" (80%); being "over the dose or limit" (39%); mixing heroin with pills or alcohol (45%); and not knowing the purity or quality of heroin (23%). The heart was commonly believed to be the part of the body primarily affected by heroin overdose (60%). Withdrawing blood in the event of overdose was common, with 38 percent reporting that they would withdraw blood in the event of an overdose. The most frequently cited justification for withdrawing blood in the event of overdose was the belief that it removes the drug or reduces the dose (53%).

As the data presented here indicate, the interpretive framework used by ethnic Vietnamese IDUs to make sense of overdose is not based on supernatural or personalistic theories and is thus amenable to, and consistent with, public health approaches. Withdrawing blood represents a rational, adaptive emic response to heroin overdose among this largely south Vietnamese immigrant population. As illustrated by participants, causal explanations for heroin overdose are impersonal and are believed to relate to conditions, such as the effects of drugs on the heart, that can be modified or prevented. Participants' beliefs about overdose, especially beliefs in the effects of drugs on the circulatory system, and the practice of withdrawing blood in response, illustrate the gap between lay Vietnamese and biomedical explanatory models of overdose. However, calling an ambulance does not challenge ethnic Vietnamese IDUs' understandings of the etiology of overdose and the practice of withdrawing blood does not present an immediate threat to the victim's health. These two very different responses to overdose, while emanating from distinctive explanatory models, are not mutually exclusive. It is the delay or avoidance of calling an ambulance in this situation that results in potentially fatal outcomes. In order to be effective, it is necessary to present this information to Vietnamese IDUs in ways which demonstrate an understanding and accommodation of emic explanatory models. Ethnic Vietnamese IDUs could be encouraged to call an ambulance and attempt to re-establish breathing rhythms prior to withdrawing blood. In addition, consideration should be given to providing Vietnamese IDUs with access to Naloxone to be administered in the event of overdose. Indeed, the qualitative data presented here suggest that the administration of an antidote may be consistent with the explanatory model of overdose employed by Vietnamese IDUs and the preference for self-managed care observed in this group.

To be effective, overdose prevention messages also need to take account of culturally specific understandings of blood and the (overdosed) body. Overdose prevention messages based on the "save-a-mate" philosophy [37] may have potential to appeal to close social networks of Vietnamese IDUs by capitalising on their strong sense of obligation to each other. However, the qualitative data presented here provide little evidence that messages such as "save-a-mate" and education campaigns which highlight the dangers of concurrent alcohol and benzodiazepine use actually reach, or are taken up by, this group. As this article has shown, there is a need for targeted overdose prevention strategies which build on, or at least take account of, existing interpretive frameworks among ethnic Vietnamese IDUs.

The data presented here illustrate the ways in which cultural beliefs and values shape health logics and practices. Different explanatory models or understandings of overdose mechanisms give rise to differences in management and response. In relation to the explanatory model employed by Vietnamese IDUs, these differences may result in preventable deaths. Thus the data on heroin overdose and the management practices of ethnic Vietnamese IDUs presented here provide a particularly dramatic example of the need for public health interventions and responses based on culturally specific meanings and contexts of health, illness and risk.

## Notes

1. While traditional Chinese and traditional Vietnamese medicine differ in practice, they share the same theoretical foundations. During the 17th century, traditional Vietnamese, Chinese and practitioners from other ethnic groups began identifying their medicine as Eastern medicine or Dong Y to differentiate it from Western colonial medicine. In this paper, Dong Y is used to refer to both Chinese and Vietnamese traditional medicine.

## Competing interests

The authors declare that they have no competing interests.

## Authors' contributions

LM conceived of the study, participated in its design and coordination, conducted ethnographic fieldwork and drafted the manuscript. HH conducted ethnographic fieldwork and in-depth interviews and helped to revise the manuscript.

## Supplementary Material

Additional file 1 **Young woman's arms following withdrawal of blood subsequent to heroin overdose**. Image. Click here for file

## References

[B1] Darke S, Degenhardt L, Mattick R (2006). Mortality amongst illicit drug users: Epidemiology, causes and intervention.

[B2] Hulse GK, English DR, Milne E, Holman CDJ (1999). The quantification of mortality resulting from the regular use of illicit opiates. Addiction.

[B3] Darke S, Ross J, Zador D, Sunjic S (2000). Heroin-related deaths in New South Wales, Australia, 1992–1996. Drug and Alcohol Dependence.

[B4] Warner-Smith M, Darke S, Lynskey M, Hall W (2001). Heroin overdose: Causes and consequences. Addiction.

[B5] Darke S, Kaye J, Duflou J (2006). Systemic disease among cases of fatal opioid toxicity. Addiction.

[B6] Drew LRH (1982). Avoidable deaths from drug intoxification. Medical Journal of Australia.

[B7] Garriot JC, Sturner WQ (1973). Morphine concentrations and survival periods in acute heroin fatalities. New England Journal of Medicine.

[B8] Zador D, Sunjic S, Darke S (1996). Heroin-related deaths in NSW, 1992: Toxicological findings and circumstances. Medical Journal of Australia.

[B9] Barr A, Crofts N (1998). Vietnamese drug related deaths in Victoria 1992–1997.

[B10] Maher L, Sargent PL, Higgs P, Crofts N, Kelsall J, Le T (2001). Risk behaviours of young Indo-Chinese injecting drug users in Sydney and Melbourne. Australian and New Zealand Journal of Public Health.

[B11] Ho HT (2006). Culture, risk and vulnerability to blood-borne viruses among ethnic Vietnamese injecting drug users. PhD thesis.

[B12] Darke S, Ross J (1998). Heroin-related death in South Western Sydney, Australia. Drug and Alcohol Review.

[B13] Maher L, Dixon D (1999). Policing and public health: Law enforcement and harm minimization in a street-level drug market. British Journal of Criminology.

[B14] Reid G, Higgs P, Beyer L, Crofts N (2002). Vulnerability among Vietnamese illicit drug users in Australia: challenges for change. International Journal of Drug Policy.

[B15] Louie R, Krouskos D, Gonzalez M, Crofts N Vietnamese-speaking injecting drug users in Melbourne: The need for harm reduction. Australian and New Zealand Journal of Public Health.

[B16] Maher L, Chant K, Jalaludin B, Sargent PL (2004). Risk behaviours and antibody hepatitis B and C prevalence among injecting drug users in South Western Sydney, Australia. Journal of Gastroenterology and Hepatology.

[B17] Hellard ME, Nguyen OK, Higgs PG, Guy RJ, Jardine D, Mijch A (2007). The prevalence and risk behaviours associated with the transmission of blood borne viruses among ethnic Vietnamese injecting drug users in Victoria. Australian and New Zealand Journal of Public Health.

[B18] Beyer L, Reid G, Crofts N (2001). Ethnic based differences in drug offending. Australian and New Zealand Journal of Criminology.

[B19] Ezard N, Ritter A, Kutin J, Lintzeris N, Bammer G (1997). Trialling the new pharmacotherapies in the Vietnamese community: appropriateness and possible options. Expanding treatment options for heroin dependence in Victoria: buprenorphine, LAAM, naltrexone and slow-release oral morphine.

[B20] Dunlop AJ (2006). Tro Choi Moi: a study of ethnic Vietnamese heroin users and the use of buprenorphine for withdrawal and post withdrawal treatment. PhD thesis.

[B21] Kleinman A (1980). Patients and healers in the context of culture.

[B22] Maher L, Coupland H, Musson R (2007). Scaling up HIV treatment, care and support for injecting drug users in Vietnam. International Journal of Drug Policy.

[B23] Maher L, Swift W, Dawson M (2001). Heroin purity and composition in Sydney, Australia. Drug and Alcohol Review.

[B24] Ho HT, Maher L (2008). Co vay co tra: Culture, risk and vulnerability to blood borne viruses among ethnic Vietnamese injecting drug users. Drug and Alcohol.

[B25] Glaser BG, Strauss AL (1967). The discovery of grounded theory: Strategies for qualitative research Chicago, Aldine.

[B26] Biernacki P, Waldorf D (1982). Snowball sampling: Problems and techniques of chain referral sampling. Social Methods & Research.

[B27] Ezzy D (2001). Qualitative analysis.

[B28] Hanoi Medical University (2000). Y hoc co truyen [Traditional medicine].

[B29] Ping C Concepts and theories of traditional Chinese medicine.

[B30] American Psychiatric Association (1994). Diagnostic and statistical manual of mental disorders: DSM-IV.

[B31] Hoang GN, Erickson RV (1985). Cultural barriers to effective medical care among Indo-Chinese patients. Annual Review of Medicine.

[B32] Seaman SR, Brettle RP (1998). Mortality from overdose among injecting drug users recently released from prison: Database linkage study. British Medical Journal.

[B33] Coomber R (1997). The adulteration of drugs: What dealers do to illicit drugs, and what they think is done to them. Addiction Research.

[B34] Coomber R, Maher L (2006). Street-level drug market activity in Sydney's primary heroin markets: Organisation, adulteration practices, pricing, marketing and violence. Journal of Drug Issues.

[B35] White JM, Irvine RJ (1999). Mechanism of fatal opioid overdose. Addiction.

[B36] Darke S, Zador D (1996). Fatal heroin "overdose": A review. Addiction.

[B37] Australian Red Cross Save – A – Mate (SAM).

