# Supplementary figures and images for: Overdose beliefs and management practices among ethnic Vietnamese heroin users in Sydney, Australia
Source: Harm Reduct J. 2009 Apr 27;6:6. doi: 10.1186/1477-7517-6-6 (PMC2679730; doi:10.1186/1477-7517-6-6)

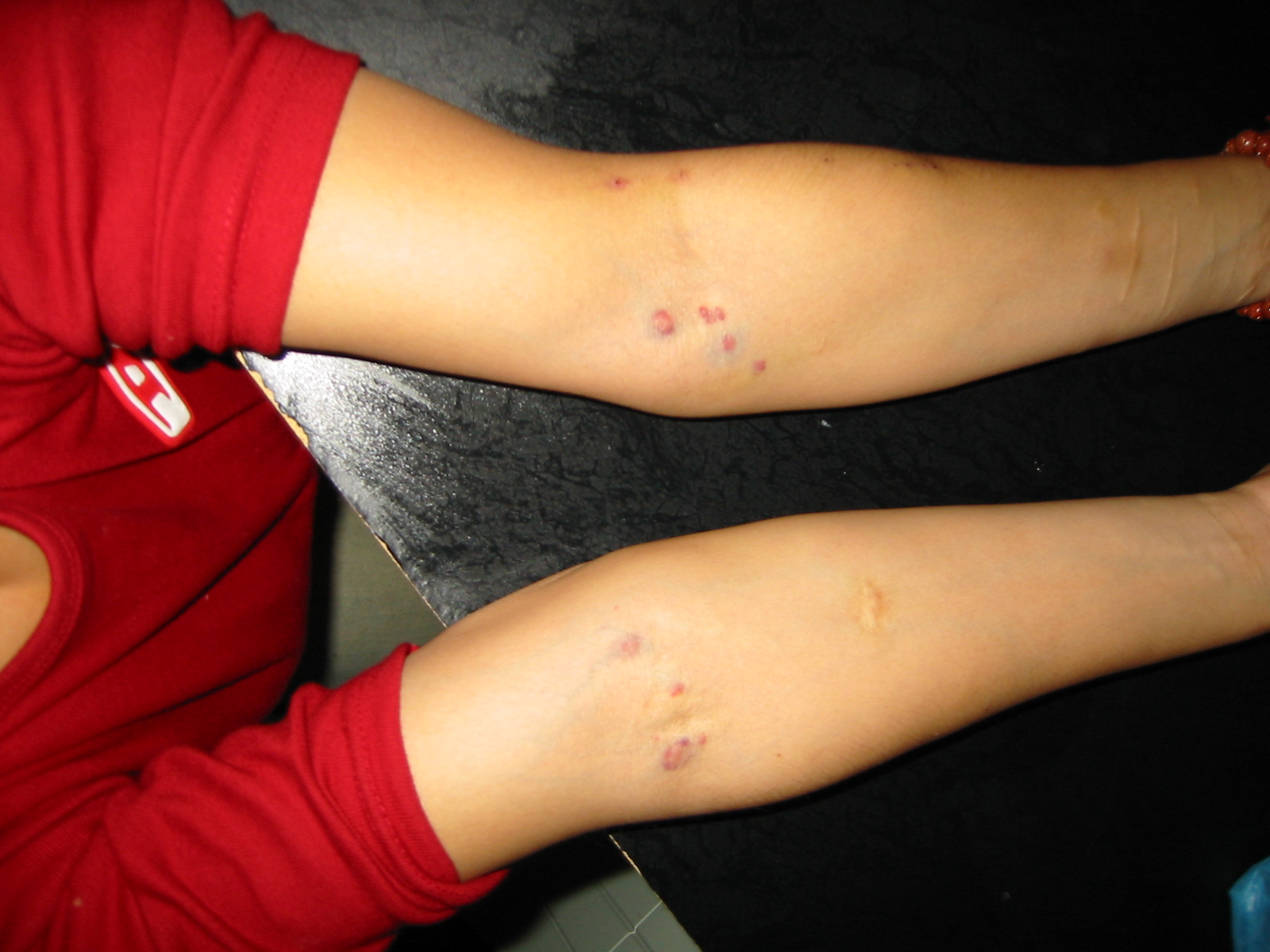

Supplement: Additional file 1 — Young woman's arms following withdrawal of blood subsequent to heroin overdose. Image. [file 1477-7517-6-6-S1.jpeg]
